# Na^+^/Ca^2+^ exchanger mediates cold Ca^2+^ signaling conserved for temperature-compensated circadian rhythms

**DOI:** 10.1126/sciadv.abe8132

**Published:** 2021-04-30

**Authors:** Naohiro Kon, Hsin-tzu Wang, Yoshiaki S. Kato, Kyouhei Uemoto, Naohiro Kawamoto, Koji Kawasaki, Ryosuke Enoki, Gen Kurosawa, Tatsuto Nakane, Yasunori Sugiyama, Hideaki Tagashira, Motomu Endo, Hideo Iwasaki, Takahiro Iwamoto, Kazuhiko Kume, Yoshitaka Fukada

**Affiliations:** 1 Department of Biological Sciences, School of Science, The University of Tokyo, Hongo 7-3-1, Bunkyo-ku, Tokyo 113-0033, Japan.; 2 Department of Neuropharmacology, Graduate School of Pharmaceutical Sciences, Nagoya City University, Nagoya 467-8603, Japan.; 3 Graduate School of Biological Science, Nara Institute of Science and Technology, Ikoma 630-0192, Japan.; 4 Graduate School of Biostudies, Kyoto University, Kyoto 606-8501, Japan.; 5 Department of Electrical Engineering and Bioscience, Waseda University, Tokyo 162-8480, Japan.; 6 Biophotonics Research Group, Exploratory Research Center on Life and Living Systems (ExCELLS), National Institutes of Natural Sciences, Higashiyama 5-1, Myodaiji, Okazaki, Aichi 444-8787, Japan.; 7 Division of Biophotonics, National Institute for Physiological Sciences, National Institutes of Natural Sciences, Higashiyama 5-1, Myodaiji, Okazaki, Aichi 444-8787, Japan.; 8 iTHEMS, RIKEN, Wako 351-0198, Japan.; 9 Department of Life Sciences, Faculty of Agriculture, Kagawa University, Kagawa 761-0795, Japan.; 10 Department of Pharmacology, Faculty of Medicine, Fukuoka University, Fukuoka 814-0180, Japan.

## Abstract

Circadian rhythms are based on biochemical oscillations generated by clock genes/proteins, which independently evolved in animals, fungi, plants, and cyanobacteria. Temperature compensation of the oscillation speed is a common feature of the circadian clocks, but the evolutionary-conserved mechanism has been unclear. Here, we show that Na^+^/Ca^2+^ exchanger (NCX) mediates cold-responsive Ca^2+^ signaling important for the temperature-compensated oscillation in mammalian cells. In response to temperature decrease, NCX elevates intracellular Ca^2+^, which activates Ca^2+^/calmodulin-dependent protein kinase II and accelerates transcriptional oscillations of clock genes. The cold-responsive Ca^2+^ signaling is conserved among mice, *Drosophila*, and *Arabidopsis*. The mammalian cellular rhythms and *Drosophila* behavioral rhythms were severely attenuated by NCX inhibition, indicating essential roles of NCX in both temperature compensation and autonomous oscillation. NCX also contributes to the temperature-compensated transcriptional rhythms in cyanobacterial clock. Our results suggest that NCX-mediated Ca^2+^ signaling is a common mechanism underlying temperature-compensated circadian rhythms both in eukaryotes and prokaryotes.

## Teaser

NCX is a new clock component common to eukaryotes and prokaryotes.

## INTRODUCTION

Among a wide variety of biological functions, the circadian clock is of particular interest because of its unique property, i.e., temperature-compensated oscillation with a period of approximately 24 hours (*1*). Generally, an increase in temperature by 10°C accelerates rates of biochemical reactions by two- to threefold (*Q*_10_ = 2 to 3), whereas *Q*_10_ of the oscillation speed of the clock is 0.8 to 1.2. The property was originally termed temperature independence, but later termed temperature compensation on the basis of the finding of overcompensation for the effect of temperature on the period length in photosynthetic dinoflagellates (*Lingulodinium polyedra*) (*1*). The temperature compensation is a common property of the circadian clocks, implicating that a mechanism underlying the compensation is tightly associated with machinery for cell-autonomous oscillation.

Most of the overt circadian rhythms are based on biochemical oscillations generated by clock genes and their encoded proteins (*2*–*5*). Homologies of the clock genes are limited among animals, fungi, plants, and cyanobacteria, suggesting that the clock genes independently evolved after divergence of the lineages. In cyanobacteria, KaiC phosphorylation rhythms constitute a core circadian oscillator termed posttranslational oscillator (PTO) (*5*). The phosphorylation rhythms of KaiC in the KaiA-KaiB-KaiC protein complex are temperature compensated in vitro. In eukaryotes, clock genes and their encoded proteins constitute transcriptional/translational feedback loops (TTFLs) (*2*–*4*). Because a HES (Hairy and Enhancer of Split)–based TTFL in segmentation clock is temperature sensitive (*Q*_10_ = 2 to 3) (*6*), temperature compensation is not a general property intrinsic to TTFLs. This suggests the existence of an important mechanism regulating the circadian oscillation of the TTFLs.

Historically, before the discovery of the clock genes, a feedback system involving ions and ion regulators in plasma membranes was proposed as the oscillation mechanism of the circadian clock (*7*). This “membrane model” is based on the observation that the circadian rhythms are notably affected by manipulating ion concentrations or ion regulator activities in various eukaryotes (*7*). To date, several ions, especially Ca^2+^, have been shown to play an essential role for oscillation of the TTFLs in mammals (*8*), insects (*9*), and plants (*10*). In mice and *Drosophila*, intracellular Ca^2+^ levels were shown to exhibit robust circadian oscillations (*11*–*13*), which elicit rhythmic activation of Ca^2+^/calmodulin-dependent protein kinase II (CaMKII) (*14*–*16*). CaMKII phosphorylates CLOCK to activate CLOCK-BMAL1 heterodimer, a key transcriptional activator in the animal TTFLs (*3*). The upstream regulator of the Ca^2+^-dependent phosphorylation signaling has been a missing link between the TTFL and the membrane model.

## RESULTS

### Ca^2+^ signaling is a key for temperature-compensated oscillation

**Fig. 1:**
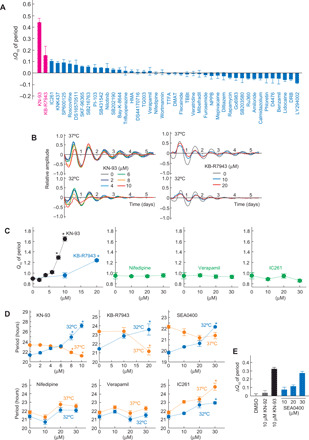
CaMKII and NCX activities are essential for temperature compensation. (**A**) Effects of chemical inhibitors on *Q*_10_ of bioluminescence rhythms in Rat-1–*Bmal1*-luc cells. To normalize experiment-to-experiment variations, Δ*Q*_10_ values compared to the vehicle (DMSO) control were used for comparison of the all screening data. The waveforms and the other parameters are shown in figs. S1 to S3. (**B**) Representative bioluminescence rhythms of Rat-1–*Bmal1*-luc cells in the presence of KN-93 (left) or KB-R7943 (right) at 32° or 37°C. (**C**) Dose-dependent effect of KN-93 or KB-R7943 on *Q*_10_. ★*P* < 0.05 compared to DMSO (Dunnett’s test). (**D**) Dose- and temperature-dependent effect of KN-93, KB-R7943, or SEA0400 on period length at 32° or 37°C. ★*P* < 0.05 compared to DMSO (Dunnett’s test). (**E**) Effect of KN-92, KN-93, or SEA0400 on Δ*Q*_10_ value. We used a concentration of 10 μM consistently in the first screening (A), and two compounds, KN-93 and KB-R7943, met our criteria. Then, we performed reproducibility test and dose dependency test for the two compounds with several control compounds (B to E). Representative data (B) or the means with SEM from three independent samples (A and C to E) are shown.

To uncover key regulators involved in temperature-compensated oscillation in mammals, we investigated effects of various small-molecule compounds targeting protein kinases or ion regulators (table S1) on cellular rhythms of Rat-1 fibroblasts stably expressing *Bmal1*-luciferase reporter (*14*, *17*). A *Q*_10_ value was calculated from the period lengths of the bioluminescence rhythms recorded at 32° and 37°C (figs. S1, A and B, and S2A). All the screening results were evaluated by using a Δ*Q*_10_ value, which was defined as a difference of the *Q*_10_ values between drug-treated cells and control [0.1% dimethyl sulfoxide (DMSO)–treated] cells (Fig. 1A). We found a remarkable increase in Δ*Q*_10_ by CaMKII inhibitor KN-93 (Fig. 1A) in a dose-dependent manner (Fig. 1, B and C). The treatment with 10 μM KN-93 shortened the period at 37°C, whereas it lengthened the period at 32°C (Fig. 1D). Such a temperature-dependent bidirectional effect of KN-93 was unique in that many compounds showed a unidirectional period-modifying effect at 32° and 37°C (fig. S1C). KN-92, an inactive analog of KN-93, had no significant effect on Δ*Q*_10_ value (Fig. 1E and fig. S2B), supporting the specific effect of KN-93 on CaMKII.

In detailed analysis of the effects of the compounds, we noticed that the treatment with KB-R7943, an inhibitor of Na^+^/Ca^2+^ exchanger (NCX) (*18*), increased the *Q*_10_ value in a dose-dependent manner (Fig. 1, B and C). Similar to the CaMKII inhibitor, KB-R7943 exhibited the temperature-dependent bidirectional effect on the circadian period (Fig. 1D). Another NCX inhibitor, SEA0400 (*18*), also showed the bidirectional period-modifying effect (Fig. 1D) and the *Q*_10_-increasing effect (Fig. 1E). On the other hand, none of these effects were observed after treatment of Rat-1 cells with nifedipine and verapamil, blockers of L-type Ca^2+^ channel, or with IC261, a period-lengthening inhibitor of casein kinase I (Fig. 1, C and D, and fig. S3) (*15*).

**Fig. 2:**
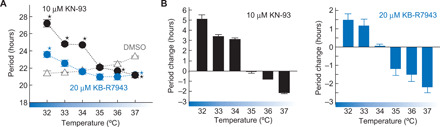
Temperature compensation is compromised by CaMKII or NCX inhibitor. (**A**) Period length of Rat-1–*Bmal1*-luc cells in the presence of KN-93 or KB-R7943 at 32°, 33°, 34°, 35°, 36°, or 37°C. ★*P* < 0.05 compared to DMSO (Student’s *t* test). (**B**) Temperature-dependent effect of KN-93 or KB-R7943 on the period length. The means with SEM from three independent samples (A and B) are shown.

The period-modifying effects of KN-93 and KB-R7943 were further analyzed at various temperatures between 32° and 37°C (fig. S4). As a control, Rat-1 cells treated with DMSO showed shorter periods at lower temperatures (Fig. 2A), a phenomenon termed overcompensation observed in a wide range of species (*1*–*5*). In contrast, the oscillation speed was slowed down by decreasing the temperature in the presence of KN-93 or KB-R7943 (Fig. 2A), and this period-lengthening effect was particularly obvious below 35°C (Fig. 2B). The *Q*_10_ value calculated from the circadian periods at 32° and 35°C was 0.89 (vehicle), 1.49 (20 μM KB-R7943), or 2.01 (10 μM KN-93). It is evident that the overcompensated oscillation becomes temperature sensitive by inhibiting CaMKII or NCX activity. These results together demonstrate that CaMKII and NCX are key players for temperature compensation in the mammalian cellular clock.

### NCX-Ca^2+^-CaMKII signaling is important for cellular circadian oscillation

**Fig. 3:**
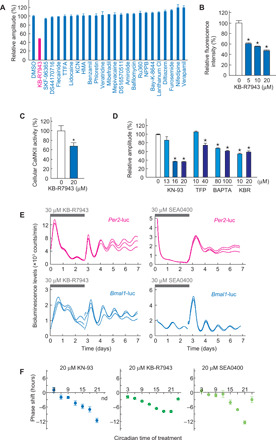
NCX-dependent Ca^2+^-CaMKII signaling is a key determinant of the state of circadian oscillator. (**A**) Effects of various ion channel modulators on the amplitude of Rat-1–*Bmal1*-luc cells. Bioluminescence rhythm data are shown in fig. S2. (**B**) Effects of NCX inhibitors on intracellular Ca^2+^ levels. The Ca^2+^ level changes by the drug were measured by using Fluo-4 in NIH3T3 cells. ★*P* < 0.05 compared to DMSO (Dunnett’s test). (**C**) Effects of the NCX inhibitors on intracellular CaMKII levels in NIH3T3 cells. After 1-day treatment with the inhibitor or DMSO, phosphorylation activity of the cell lysate was measured with syntide-2. ★*P* < 0.05 compared to DMSO (Student’s *t* test). (**D**) Effects of Ca^2+^-CaMKII signaling inhibitors on amplitude of the rhythms in Rat-1–*Bmal1*-luc cells. ★*P* < 0.05 compared to DMSO (Dunnett’s test). The level of DMSO control was set to 100% (A to D). TFP, trifluoperazine. (**E**) Reversible effects of NCX inhibitors on bioluminescence rhythm of Rat-1–*Bmal1*-luc cells or Rat-1–*Per2*-luc cells. (**F**) Effects of pulse inhibition of CaMKII or NCX on phase of the oscillator. Two-hour treatment of KN-93 (left) or KB-R7943 (middle) or 1-hour treatment of SEA0400 (right) was applied to Rat-1–*Bmal1*-luc cells at various circadian time (CT). CT12 was defined as trough level of *Bmal1*-luc rhythm. Because the treatment of KN-93 at CT24 resulted in the disappearance of the cellular rhythm, the extent of the phase shift was not determined. Data shown are means with SEM from three (A, B, D, and F) or eight (C) independent samples. All experimental data in this figure were obtained from cells cultured at 37°C. nd, not determined.

Note that the KB-R7943 treatment of Rat-1 fibroblasts decreased the amplitude of the cellular rhythms (Fig. 1B). Among the chemicals targeting ion channels and transporters, only KB-R7943 suppressed the relative amplitude of the rhythms (Fig. 3A), suggesting an important role of NCX in the cell-autonomous oscillation mechanism, in addition to the temperature compensation.

NCX exchanges 3 Na^+^ for 1 Ca^2+^ across the plasma membrane. NCX is a unique bidirectional regulator of cytosolic Ca^2+^ concentration because it can mediate both Ca^2+^ influx and efflux, depending on not only the membrane potential but also local concentrations of Na^+^ and Ca^2+^ (*18*). In response to an increase in cytoplasmic Ca^2+^ levels, NCX mediates Ca^2+^ efflux, while NCX can maintain steady-state levels of intracellular Ca^2+^ by promoting Ca^2+^ influx in several types of cells (*18*). We examined roles of NCX in regulation of intracellular Ca^2+^ levels in NIH3T3 fibroblasts. Fluo-4 acetoxymethyl ester (Fluo-4 AM)–based Ca^2+^ imaging revealed that the basal fluorescence level in the cultured cells was remarkably reduced by the addition of 5 to 20 μM NCX inhibitor KB-R7943 to the culture medium (Fig. 3B), indicating that NCX contributes to net Ca^2+^ influx in the quiescent state. Then, we evaluated the effect of KB-R7943 on cellular CaMKII activity, which reflects intracellular Ca^2+^ level (*14*). One-day treatment of NIH3T3 cells with 20 μM KB-R7943 significantly decreased the CaMKII activity toward syntide-2, a model substrate specific to CaMKII (Fig. 3C) (*14*). These results reveal an important role of NCX in the maintenance of the activity level of Ca^2+^-CaMKII signaling in the fibroblasts.

To address the role of NCX-Ca^2+^-CaMKII signaling in cell-autonomous oscillation, we investigated effects of chronic inhibition of the signaling on circadian rhythms in Rat-1 reporter cells (*14*, *17*). The relative amplitude of the cellular bioluminescence rhythm detected by *Bmal1*-luc was markedly reduced by chronic treatment with KN-93, trifluoperazine (calmodulin antagonist), 1,2-bis(2-aminophenoxy)ethane-*N*,*N*,*N*′,*N*′-tetraacetic acid (BAPTA)–AM (intracellular Ca^2+^ chelator), or KB-R7943 (Fig. 3D). The amplitude-reducing effect by chronic treatment with KB-R7943 or SEA0400 was also observed in *Per2*-luc reporter cells (Fig. 3E). The severe damping of the transcriptional rhythms was reversed by washing out the drug-containing medium (Fig. 3E). On the other hand, a pulse treatment (for 1 to 2 hours) of Rat-1–*Bmal1*-luc cells with KN-93, KB-R7943, or SEA0400 caused a phase-dependent phase shift of the bioluminescence rhythms with their maximal responses at circadian time (CT)21 (Fig. 3F). The amplitude-reducing effects (Fig. 3, D and E) and the overt phase-resetting actions of the inhibitors (Fig. 3F) together suggest that NCX-dependent Ca^2+^-CaMKII signaling functions as a state variable of the circadian oscillator in a limit cycle interpretation (fig. S5) (*14*, *19*).

### Lowering temperature activates NCX-Ca^2+^-CaMKII signaling

**Fig. 4:**
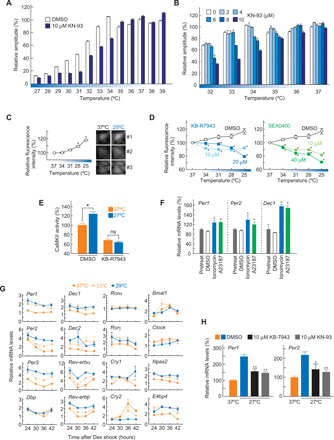
Hypothermic activation of NCX-dependent Ca^2+^-CaMKII signaling. (**A**) Effects of temperature on amplitude of cellular rhythm in Rat-1–*Bmal1*-luc cells. (**B**) Temperature- and dose-dependent effects of KN-93 on amplitude of cellular rhythm in Rat-1–*Bmal1*-luc cells. (**C**) Hypothermic Ca^2+^ response in NIH3T3 cells. The mean value at 37°C is set to 100. ★*P* < 0.05 compared to 37°C (Dunnett’s test). Right panels are representative images of intracellular Ca^2+^ levels in NIH3T3 cells at 37° or 25°C. (**D**) KB-R7943 or SEA0400 blocks hypothermic Ca^2+^ response in NIH3T3 cells. Initial value of each cell at 37°C was set to 100%. ★*P* < 0.5 × 10^−7^ compared to DMSO (Student’s *t* test). The Ca^2+^ imaging analysis was started from 37°C down to 25°C (C and D). (**E**) NCX mediates hypothermic CaMKII activation in NIH3T3 cells. The mean value of DMSO at 37°C is set to 100%. ★*P* < 0.05 (Student’s *t* test). ns, not significant. (**F**) Ca^2+^ ionophore up-regulates clock gene *Per1*, *Per2*, and *Dec1*. Thirty-six hours after the rhythm induction with dexamethasone, 10 μM (final concentration) ionomycin, A23187, or the same volume of DMSO was applied to Rat-1–*Bmal1*-luc cells. One hour after the treatment, the cells were harvested to detect clock gene mRNA levels. The mean value of pretreatment is set to 100%. ★*P* < 0.05 compared to DMSO (Student’s *t* test). (**G**) Hypothermic response of clock genes in Rat-1–*Bmal1*-luc cells. (**H**) NCX and CaMKII mediate hypothermic up-regulation of *Per1* and *Per2* in Rat-1–*Bmal1*-luc cells. The mean value at 37°C is set to 100%. ★*P* < 0.05 and ★★*P* < 0.005 compared to DMSO-treated cells at 27°C (Student’s *t* test). The cells were harvested to detect clock gene mRNA levels at indicated time points (G) or 5 days (H) after rhythm induction by dexamethasone. Representative data [panels of (C)] or means with SEM from 3 (A, B, F, and G), 8 (E), 9 (H), or 20 (C and D) independent samples are shown.

In the experiments examining the relationship between temperature and the cellular rhythms, we found that the amplitude of the rhythm was decreased by lowering temperature particularly below 34°C (Fig. 4A, DMSO, and fig. S6). In the same range of temperatures, KN-93 treatment (2 to 10 μM) caused a much larger decrease in the amplitude in a dose-dependent manner (Fig. 4, A and B, and fig. S6). The results indicate that CaMKII activity compensates for amplitude decrease in the TTFL at temperatures below 34°C.

Note that hypothermia is clinically defined as a drop in core body temperature below 35°C (*20*). We hypothesized that Ca^2+^ signaling may be activated for cold response in mammalian cells, as reported for cold tolerance mechanism of insects and plant cells (*21*). This idea was tested by investigating intracellular Ca^2+^ levels in cultured fibroblasts. In Fluo-4 AM–based Ca^2+^ imaging, lowering of the temperature from 37° to 25°C significantly increased free Ca^2+^ levels in NIH3T3 cells (*Q*_10_ = 0.73) (Fig. 4C). The hypothermic response was blocked by treatment with SEA0400 or KB-R7943 (Fig. 4D). We found that the CaMKII activity of lysates prepared from the cells cultured at 27°C was higher than that at 37°C (*Q*_10_ = 0.78) (Fig. 4E, DMSO). In addition, the hypothermic activation of CaMKII was inhibited in the cells cultured with 20 μM KB-R7943 (Fig. 4E). These results indicate that NCX enhances Ca^2+^ influx and activates CaMKII signaling in response to the temperature decrease in the mammalian cells.

We then examined how intracellular Ca^2+^ levels affect the clock gene expression rhythms. In Rat-1–*Bmal1*-luc cells, 1-hour treatment with Ca^2+^ ionophore, ionomycin, or A23187 up-regulated transcripts of *Per1*, *Per2*, and *Dec1* (Fig. 4F), which are regulated by CaMKII (*14*, *16*) through E-box and/or CRE, a DNA cis-element responsive to Ca^2+^/cyclic adenosine monophosphate signaling (*3*). Consistently, a decrease in the temperature from 37°C down to 29°C elevated the expression levels of many E-box–regulated genes, such as *Per1*, *Per2*, *Per3*, *Dec1*, *Dec2*, *Rev-erb*α, *Rev-erb*β, and *Cry1* (Fig. 4G). In addition, *E4bp4*, which is regulated by Ca^2+^-NFAT signaling (*22*), is also up-regulated by the temperature decrease (Fig. 4G). Consistent with the decrease in relative amplitude of the bioluminescence rhythms at lower temperatures (Fig. 4A), a peak-trough ratio of *Bmal1* expression rhythm was reduced by lowering the temperature (Fig. 4G). We found that the hypothermic up-regulation of *Per1* and *Per2* transcripts was significantly attenuated in the presence of NCX inhibitor KB-R7943 or CaMKII inhibitor KN-93 (Fig. 4H). These results together indicate that the temperature changes have a marked influence on the clock gene expression levels through NCX-Ca^2+^-CaMKII signaling.

### Cold-responsive Ca^2+^ signaling compensates for slowdown of TTFL at lower temperature

**Fig. 5:**
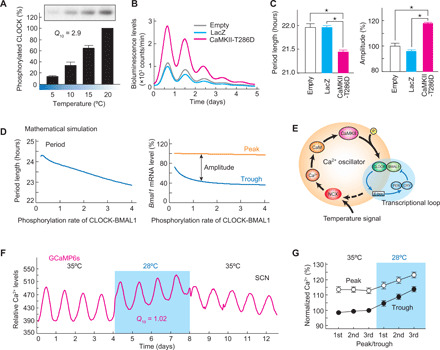
Mechanism of temperature compensation. (**A**) Effect of temperature on phosphorylation activity of purified CaMKII. Phosphorylation activity of rat CaMKII against CLOCK peptide was measured by autoradiography. The level of phosphorylated CLOCK at 20°C was set to 100%. (**B**) Effect of CaMKII overexpression on bioluminescence rhythm by *Bmal1*-luc in NIH3T3 cells. (**C**) Effect of CaMKII overexpression on period length and amplitude of cellular rhythm. ★*P* < 1.0 × 10^−6^ (Student’s *t* test with Bonferroni correction). (**D**) Mathematical simulation of effect of CLOCK-BMAL1 activation on period length and amplitude of *Bmal1* expression rhythms. (**E**) Circadian Ca^2+^ oscillator regulates TTFL to generate temperature-compensated overt rhythms in mammalian circadian clock. (**F**) Effect of temperature on Ca^2+^ oscillation in SCN. (**G**) Hypothermia increases trough and peak levels of Ca^2+^ oscillation in SCN. The fluorescence levels of GCaMP6s were divided by those of mRubby for normalization of effect of temperature on the fluorescence indicator. Representative data [top panels of (A), (B) and (F)] or means with SEM (A, C, and G) from three (A), four (B and C), or seven (F and G) independent samples are shown.

In 1957, Hastings and Sweeney (*1*) hypothesized that temperature compensation of the circadian clock is based on a combination of temperature-sensitive period-shortening and period-lengthening processes. Most biochemical reactions in the TTFL are slowed down by decreasing the temperature (table S2). In an in vitro assay, kinase activity of purified CaMKII toward a CLOCK peptide (Ser/Pro-rich region of CLOCK) (*15*) was reduced by lowering the temperature (*Q*_10_ = 2.9) (Fig. 5A). In contrast, as described above (Fig. 4E), CaMKII activity in the cultured cells was enhanced by lowering the temperature (*Q*_10_ = 0.78), indicating that Ca^2+^ influx is a key factor for accelerating CaMKII-mediated processes in the circadian clock at lower temperatures. Overexpression of CaMKIIα-T286D, a constitutive active form of CaMKIIα (*14*), accelerated the oscillation speed (shortened the period) and increased the amplitude of *Bmal1*-luc rhythms in cultured NIH3T3 cells (Fig. 5, B and C). To understand the experimental results theoretically, we simulated the effect of phosphorylation-dependent activation of CLOCK-BMAL1 on the gene expression rhythm by using a previously published mathematical model (*23*). In the horizontal axis of this simulation (Fig. 5D), a standard phosphorylation rate of CLOCK-BMAL1 estimated from previous experimental results was set to 1 (*23*). We found that an increase in the phosphorylation rate of CLOCK-BMAL1 accelerated the oscillation speed (shortened the period) and increased the amplitude of *Bmal1* mRNA rhythm (Fig. 5D). These theoretical analysis and experimental data collectively indicate that the cold-responsive Ca^2+^ signaling compensates for the period lengthening and amplitude reduction of the TTFL caused by lowering the temperatures. Considering the roles of intracellular Ca^2+^ in the circadian oscillation of the TTFL (Fig. 3) and in its temperature compensation (Figs. 1, 2, 4, and 5, A to D), we propose an oscillation model in which the TTFL couples with a Ca^2+^ oscillator for temperature-compensated circadian rhythms (Fig. 5E). We then examined responses of the Ca^2+^ oscillator to temperature changes. Circadian rhythms of intracellular Ca^2+^ levels in cultured slices of the mouse suprachiasmatic nucleus (SCN) were monitored by using adeno-associated virus-mediated gene transfer of GCaMP6s (*11*). Lowering the temperature from 35° to 28°C caused upward shifts of both the peak and trough levels of the intracellular Ca^2+^ (Fig. 5, F and G) with no significant change in the period length of the Ca^2+^ oscillation (*Q*_10_ = 1.02). These results together suggest that the circadian Ca^2+^ oscillator is highly responsive to temperature changes to maintain constant period lengths of cellular circadian rhythms.

### Cold-responsive phosphorylation signaling is conserved among animals and plants

**Fig. 6:**
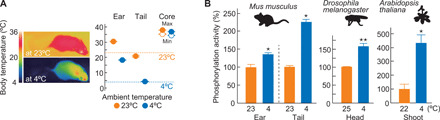
Cold-responsive phosphorylation signaling conserved in animals and plants. (**A**) Body temperature of mice at normal (23°C) or cold (4°C) temperature. Surface body temperature was measured by infrared thermography. Representative images are shown in the left panel. Core body temperature of mice was measured by implantable device in peritoneal cavity (fig. S7). (**B**) Hypothermic activation of cellular phosphorylation activity against syntide-2 in the ear or tail of *Mus musculus*, the head of *D. melanogaster*, or the shoot of *A. thaliana*. The phosphorylation activity at normal temperature was set to 100%. ★*P* < 0.01 and ★★*P* < 1.0 × 10^−5^ compared to nontreated samples (Student’s *t* test). Data are means with SEM from three independent samples (A and B).

The cold-responsive Ca^2+^ signaling was investigated in vivo in several organisms. Cold exposure of mice to 4°C for 10 min remarkably decreased the temperatures of the body surface (Fig. 6A) without a large change in the core body temperature (fig. S7A). Infrared thermography revealed that the temperatures of the ear and tail dropped by 12.0° and 16.5°C, respectively (Fig. 6A). We found that CaMKII activities (toward syntide-2) in the tissue lysates were enhanced by 1.34-fold (ear) and 2.25-fold (tail) after 90-min exposure at 4°C (Fig. 6B). The cold response of CaMKII was also analyzed in *Drosophila melanogaster*. CaMKII activities in the fly heads were enhanced by 1.57-fold (Fig. 6B), when the flies (maintained at 25°C) were exposed to 4°C for 90 min. In plants, Ca^2+^-dependent protein kinases (CDPKs) are the major transducers of Ca^2+^ signaling (*24*). Catalytic domains of CDPKs are highly homologous to animal CaMKII, and syntide-2 is a model substrate of CDPKs (*24*). The enzymatic activities phosphorylating syntide-2 in the shoot (leaf and stem) lysates of *Arabidopsis thaliana* (kept at 22°C) were remarkably enhanced by 90-min exposure at 4°C (Fig. 6B). These results suggest that activation of Ca^2+^-dependent phosphorylation signaling is a conserved mechanism underlying the cold responses.

### Roles of NCX in circadian clockworks are conserved in eukaryotes and prokaryotes

**Fig. 7:**
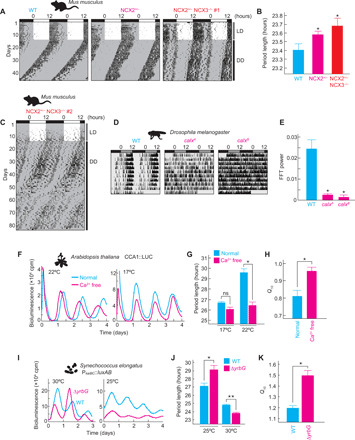
Conserved roles of Ca^2+^ signaling in circadian clockwork. (**A**) Wheel-running rhythm of NCX mutant mice. LD, light-dark; DD, constant dark.(**B**) Period length of wheel-running rhythm of the NCX mutant mice. Animal number of wild type (WT), NCX2^+/−^, or NCX2^+/−^ NCX3^−/−^ is 6, 10, or 7, respectively. ★*P* < 0.05 compared to WT (Student’s *t* test). (**C**) Aberrant pattern of morning and evening activity rhythms in NCX2^+/−^ NCX3^−/−^ mice. (**D**) Locomotor activity rhythm of *calx* mutants of *D. melanogaster*. (**E**) Relative FFT power of locomotor activity rhythm of *calx* mutants of *D. melanogaster*. Animal number of WT, *calx^A^*, or *calx^B^* is 15, 16, or 14, respectively. ★*P* < 0.5 × 10^−4^ compared to WT (Student’s *t* test). (**F**) Effect of Ca^2+^ depletion on gene expression rhythm by CCA1::LUC reporter in *A. thaliana*. (**G**) Effect of Ca^2+^ depletion on period length of gene expression rhythm by CCA1::LUC reporter in *A. thaliana*. (**H**) Effect of Ca^2+^ depletion on *Q*_10_ of gene expression rhythm by CCA1::LUC reporter in *A. thaliana*. The sample number of each group is 20. ★*P* < 1.0 × 10^−7^ (Student’s *t* test). (**I**) Effect of knockout of *yrbG* on gene expression rhythm by P*_kaiBC_::luxAB* in *Synechococcus elongatus* PCC 7942 at 25° or 30°C. (**J**) Effect of knockout of *yrbG* on period length of gene expression rhythm at 25° or 30°C. ★*P* < 0.05 and ★★*P* < 0.0005. (**K**) Effect of knockout of *yrbG* on *Q*_10_ of gene expression rhythm at 25° or 30°C. *P < 0.005. The sample number of each group is 4 (J and K). Data shown are representative (A, C, D, F, and I) or means with SEM (B, E, G, H, J, and K).

Mammalian NCXs form a multigene family composed of three members: NCX1, NCX2, and NCX3. NCX1 is ubiquitously expressed in a variety of tissues, while NCX2 and NCX3 are expressed in the brain and muscle (*18*, *25*, *26*). A previous study demonstrated that homozygous knockout of NCX1 or NCX2 results in lethality (*18*, *25*). We examined wheel-running activity rhythms of NCX2^+/−^ mice and NCX2^+/−^ NCX3^−/−^ double-mutant mice. In constant dark condition, NCX2^+/−^ and NCX2^+/−^ NCX3^−/−^ mice showed free-running rhythms with circadian periods significantly longer than that of wild-type mice (Fig. 7, A and B). One double-mutant mouse exhibited unstable coordination between onset and offset of the wheel-running activity bouts under constant darkness (Fig. 7C), a phenotype similar to that observed for CaMKIIαK42R kinase-dead knock-in mice (*14*). These results indicate that NCX2 and NCX3 play important roles in maintaining normal behavioral rhythms in mice.

In *D. melanogaster*, NCX is encoded by a single gene, *calx*. We analyzed behavioral rhythms of two different lines of *calx* mutants, *calx^A^* deficient for Na^+^/Ca^2+^ exchange currents and *calx^B^* deficient for CALX protein expression (*27*). Both *calx^A^* and *calx^B^* homozygous mutants showed severely weakened rhythmicity in locomotor activities at 25°C under constant darkness (Fig. 7D). Fast Fourier transform (FFT) analysis revealed a significant reduction in the behavioral rhythmicity in the *calx* mutants (Fig. 7, D and E), indicating an essential role of CALX in the *Drosophila* clock governing the behavioral rhythms.

Roles of Ca^2+^ in plant clocks were investigated in *A. thaliana* expressing CCA1::LUC reporter. Because *Arabidopsis* has 13 NCX genes (*28*), it is difficult to evaluate roles of NCXs genetically. Instead, we investigated effects of Ca^2+^ depletion in a growth medium on the bioluminescence rhythms. The Ca^2+^ depletion resulted in significant shortening of the free-running period in constant light condition at 22°C (Fig. 7, F and G), whereas the period-shortening effect was undetectable at 17°C. The Ca^2+^ depletion caused an increase in the *Q*_10_ value from 0.80 (in the normal medium) to 0.95 (Fig. 7H), indicating that Ca^2+^ signaling is required for accelerating the oscillation speed at lower temperatures in the plant as well.

Roles of NCX in prokaryotic circadian clocks were investigated by generating a cyanobacterial strain lacking *yrbG*, a bacterial homolog of NCX (*28*). The circadian rhythms in the Δ*yrbG* strain were monitored with P*_KaiBC_::luxAB* reporter under constant light condition (Fig. 7I). We found that *yrbG* deficiency caused significant shortening of the period length at 30°C, whereas the period was lengthened at 25°C when compared with the wild-type strain (Fig. 7, I and J). Hence, the *Q*_10_ value of the bioluminescence rhythms was increased from 1.19 to 1.49 by the depletion of *yrbG* (Fig. 7K). These results demonstrate that NCX-dependent Ca^2+^ signaling plays a conserved role in both the TTFL-based eukaryotic clock and the PTO-based prokaryotic clock systems.

## DISCUSSION

Circadian TTFLs are an elaborate system that drives a wide range of overt rhythms with various phase angles and amplitudes. The oscillation speed of the TTFLs is temperature compensated, although many of the biochemical reactions in TTFLs are slowed down by decreasing temperature (table S2). The present study demonstrates that the temperature compensation of the TTFL in mammalian cells was compromised when Ca^2+^-dependent phosphorylation signaling was inhibited (Fig. 2A). We found an important role of NCX-CaMKII activity as the state variable of the circadian oscillator (Fig. 3, D to F, and fig. S5). The present study and a series of preceding works demonstrate that the Ca^2+^ oscillator plays essential roles in the circadian oscillation mechanism (Fig. 5E) (*8*–*16*). Functional studies clearly demonstrated essential roles of NCX-dependent Ca^2+^ signaling in the three important properties of the circadian clock, i.e., cell-autonomous oscillation (Figs. 3, A to E, and 7, A to C), temperature compensation (Figs. 1, 2, 4, and 5), and entrainment (Fig. 3F). The circadian Ca^2+^ oscillation is observed in mice lacking *Bmal1* or *Cry1/Cry2* (*11*, *12*), implicating that the Ca^2+^ oscillator is an upstream regulator of the TTFL in mammals.

The effects of NCX2 and NCX3 deficiencies on the regulation of mouse behavioral rhythms (Fig. 7, A to C) suggest involvement of Na^+^/Ca^2+^ exchanging activity in the Ca^2+^ dynamics of the SCN. Previous studies showed that L-type Ca^2+^ channel (LTCC) and voltage-gated Na^+^ channel (VGSC) are required for high-amplitude Ca^2+^ rhythms in the SCN (*11*, *12*). Because NCX activities are regulated by local concentrations of Na^+^/Ca^2+^ and the membrane potential (*18*), cooperative actions of LTCC, VGSC, and NCX seem to play important roles in generation mechanism of the robust Ca^2+^ oscillations in the SCN.

**Fig. 8:**
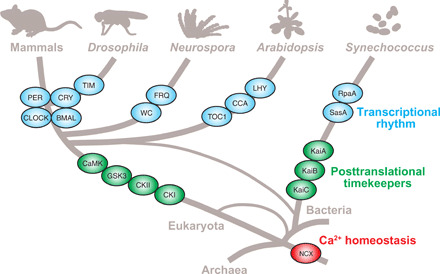
Involvement of ancient Ca^2+^ signaling for temperature-compensated circadian rhythms. Clock genes involved in the TTFLs evolved independently after divergence of each lineage. In animals, fungi, and plants, common multifunctional kinases, such as casein kinase I (CKI), CKII, glycogen synthase kinase 3 (GSK3), or Ca^2+^-dependent kinase (CaMK), are involved in posttranslational regulation of clock gene products. In cyanobacteria, posttranslational oscillator by KaiA/KaiB/KaiC drives the TTFL. NCX, a highly conserved molecule among three domains of life, is a common circadian timekeeping element in the eukaryotes and prokaryotes, and its original function is regulation of Ca^2+^ homeostasis.

It should be emphasized that the role of Ca^2+^/calmodulin-dependent protein kinases is conserved among clockworks in insects (*9*, *13*, *16*), fungi (*29*), and plants (*10*, *24*), suggesting that the Ca^2+^ oscillator might be a core timekeeping mechanism in their common ancestor (Fig. 8, Eukaryota). After divergence of each lineage, a subset of clock genes should have independently evolved in association with the Ca^2+^ oscillator. It is noteworthy that NCX is also required for temperature compensation of PTO-based cyanobacterial clock (Fig. 7, I to K). Because intracellular Ca^2+^ in cyanobacteria is elevated in response to temperature decrease (*30*), YrbG-mediated Ca^2+^ signaling may regulate the PTO in vivo. Conservation of NCX among eukaryotes, eubacteria, and archaea (Fig. 8) (*31*) suggests that NCX-dependent temperature signaling is essential for adaptation of a wide variety of organisms to environment. Further studies on NCX-regulated Ca^2+^ flux will provide evolutionary insights into the origin of the circadian clocks.

## MATERIALS AND METHODS

### Real-time monitoring of gene expression rhythms in mammalian cells

Real-time monitoring of gene expression rhythms in mammalian cells was performed by using Rat-1 fibroblasts stably expressing *Bmal1*-luciferase reporter (*14*, *15*, *17*). The fibroblasts were plated on 35-mm dishes (1.0 × 10^6^ cells per dish) and cultured at 37°C under 5% CO_2_ in a culture medium of Dulbecco’s modified Eagle’s medium (DMEM) (Sigma-Aldrich, catalog no. 5796) supplemented with 10% fetal bovine serum (FBS; Equitech-Bio Inc.), penicillin (50 U/ml), and streptomycin (50 μg/ml). One day after the plating, the cells were treated with 0.1 μM dexamethasone for 2 hours, and the medium was replaced with a recording medium of DMEM (Sigma-Aldrich, catalog no. D2902) supplemented with 10% FBS, glucose (3.5 mg/ml), penicillin (25 U/ml), streptomycin (25 μg/ml), 0.1 mM luciferin, and 10 mM Hepes-NaOH (pH 7.0). The bioluminescence signals were continually recorded from the cells cultured under air in a dish-type bioluminescence detector, Kronos (ATTO, AB-2500), or LumiCycle (Actimetrics). For overexpression of constitutive active CaMKII, pcDNA3.1-rat CaMKIIα-T286D was transfected to NIH3T3 cells expressing the *Bmal1* reporter 1 day after plating of the cells (0.5 × 10^6^ cells per 35-mm dish) (*14*). The bioluminescence rhythms from the cells were monitored (as described above) from 1 day after the transfection.

For normalization of dish-to-dish variation of the bioluminescence levels, the raw data were divided by the mean bioluminescence signals recorded for 7 days. The normalized rhythms were detrended by subtracting 24-hour centered moving averages, and the areas under the curves (arbitrary units) were used for calculating the relative amplitudes of the rhythms (*14*). Period lengths were calculated using the average value of peak-to-peak periods and trough-to-trough periods 1 day after the dexamethasone treatment of cultured cells. *Q*_10_ value was calculated by the following equation

$$Q_{10}={\left(\tau 1/\tau 2\right)}^{10/(T2‐T1)}$$

where τ1 and τ2 are the periods at temperature T1 and T2, respectively.

### Real-time monitoring of gene expression rhythms in plants

Monitoring of bioluminescence rhythms of *A. thaliana* (ecotype Columbia-0) expressing CCA1::LUC was performed as described previously (*32*). The plants were grown on a growth medium containing 10 mM KCl, 0.6 mM NH_4_NO_3_, 0.5 mM H_3_BO_3_, 0.75 mM MgSO_4_, 0.015 mM ZnSO_4_, 0.05 mM MnSO_4_, 0.05 mM FeSO_4_, 1.5 mM CaCl_2_, 0.05 mM Na_2_-EDTA, 10 mM NH_4_NO_3_, and 0.8% agar (pH 6.3) at 22°C under 12-hour light (approximately 80 μmol m^−2^ s^−1^)/12-hour dark cycles for 2 weeks. Then, the plants were transferred to the growth medium without CaCl_2_. Two days after the transfer, luciferin (final concentration of 0.125 mM) was added to the medium, and bioluminescence signals were measured with photomultiplier tubes under continuous light conditions.

### Real-time monitoring of gene expression rhythms in cyanobacteria

A strain that harbored a P*_kaiBC_::luxAB* reporter cassette with a chloramphenicol resistance gene at the targeting site (neutral site I) on the genome (ILC 976) was used as a wild-type strain. To disrupt the *yrbG* gene, a plasmid (pIL 1000) was constructed to harbor upstream and downstream regions of *yrbG* (*Synpcc7942_0242*) with a gentamicin resistance gene in the pGEM-T Easy backbone (Promega). The ∆*yrbG* strain (ILC 1383) was generated by transformation of ILC 976 with pIL 1000. Cells were grown in BG-11 media in the absence of calcium source (250 μM CaCl_2_). The bioluminescence profiles were measured with photomultiplier tubes under continuous light (LL, 40 μmol/m^2^ s) conditions after 2 days of 12-hour light/12-hour dark cycles (*33*).

### Reverse transcription polymerase chain reaction analysis

Total RNA was prepared from cultured cells using TRIzol reagent (Invitrogen) according to the manufacturer’s protocol. Reverse transcription polymerase chain reaction analysis was performed as described previously (*14*, *17*).

### Intracellular Ca^2+^ imaging

For Ca^2+^ imaging in cultured cells, NIH3T3 cells were plated on 35-mm dishes (1.0 × 10^6^ cells per dish) and cultured at 37°C under 5% CO_2_ in the culture medium. One day after the plating, the medium was replaced by an imaging buffer of Hanks’ balanced salt solution (Sigma-Aldrich, catalog no. H8264) containing 0.04% Pluronic F-127 and 1.25 mM probenecid. One hour after loading of 2 μM Fluo-4 AM at 37°C, the fluorescence intensity of the cells was monitored by a fluorescence microscope (Olympus, BX51W1) equipped with an electron multiplying charge-coupled device digital camera (Hamamatsu Photonics, C9100-13 ImagEM) in the imaging buffer. The buffer was perfused by using a peristatic pump (Gilson, MINIPULS 3) for control of the buffer temperature, which is continuously monitored by thermoelectric couple and controlled by a dual automatic temperature controller (Warner, TC-344B).

Circadian Ca^2+^ imaging in the SCN was performed as described previously (*11*). Briefly, the SCN slices were prepared from neonate mice (C57BL/6, 5 days old, both male and female). Ca^2+^ indicator protein GCaMP6s and control fluorescence protein mRuby were expressed under the control of the human synapsin-1 promoter by using adeno-associated virus (Addgene, 50942-AAV1).

### Measurement of CaMKII activity

For analysis of CaMKII activities in cultured cells, NIH3T3 cells were plated on 100-mm dishes (1.0 × 10^7^ cells per dish) and cultured at 37°C under 5% CO_2_ in the culture medium. One day after the plating, the medium was replaced by the recording medium containing the NCX inhibitor or 0.1% DMSO (vehicle), and the cells were cultured at 27° or 37°C. One day after the culture, cells were harvested by a cell scraper with 2 ml of a sampling buffer [20 mM tris-HCl, 5 mM EDTA, 1 mM EGTA, 10 mM sodium pyrophosphate, 50 mM NaF, 1 mM Na_3_VO_4_, 1 mM dithiothreitol (DTT), 0.1 mM phenylmethylsulfonyl fluoride, leupeptin (0.04 mg/ml), and aprotinin (0.04 mg/ml), pH 7.5]. For analysis of tissues, the tails or ears of C57BL/6 mice (7 weeks old, male), the heads of *D. melanogaster* (W^1118^, male), or the shoots of *A. thaliana* (ecotype Columbia-0, 14 days old) were prepared at ZT5, and 1 mg of the tissue was homogenized in 1 ml of the sampling buffer. The cells or tissues were homogenized by using a glass/Teflon homogenizer (20 strokes). CaMKII activity levels of the lysates phosphorylating syntide-2 were measured by using CaM-kinase II Assay kit (CycLex, catalog no. CY-1173) according to the manufacturer’s protocol.

For analysis of purified CaMKII activity phosphorylating a CLOCK peptide (GST-SP), CaM and rat brain CaMKII were prepared as described previously (*15*, *34*). The assay was carried out at 5°, 10°, 15°, or 20°C in a reaction mixture (10 μl) composed of 40 mM tris-HCl (pH 8.0), 2 mM DTT, 5 mM MgCl_2_, 0.5 mM CaCl_2_, 1 mM [γ-^32^P]ATP, 1 μM CaM, 100 ng of rat brain CaMKII, and 500 ng of GST-SP peptide. After incubation for 30 min, the reaction was stopped by the addition of 10 μl of 2× SDS sample buffer. Phosphorylated proteins or peptides were resolved by SDS–polyacrylamide gel electrophoresis and detected by autoradiography. We found that CaMKII activity purified from the rat brain was inactivated by incubation above 30°C, as reported by the previous study (*34*). Thus, the activity levels of the purified CaMKII were analyzed in the range of 5° to 20°C.

### Animal experiments

The animal experiments were conducted in accordance with the guidelines of the University of Tokyo. NCX2 heterozygous knockout mice (NCX2^+/−^) were produced as described previously (*25*). NCX3 homozygous knockout mice (NCX3^−/−^) were generated as follows: The targeting vector was constructed by replacing the 1.9 kilo–base pairs Eco RI–Mun I fragment containing exon 2 of the NCX3 gene with a PGK (Phosphoglycerate kinase promoter)–neo cassette. The targeted ES (embryonic stem cell) clones were confirmed by Southern blot analysis and used for the generation of germline chimeras. Chimeric male mice were crossed with female C57BL/6 mice to establish the germline transmission and backcrossed to C57BL/6 mice for more than 10 generations. The mutant mice (C57BL/6 background, male, 6 to 8 weeks old) were housed individually at 23°C in cages (13 × 23 × 15 cm) equipped with a running wheel (diameter, 10 cm) with food and water available ad libitum. Wheel-running rhythms were monitored under constant dark condition after housing under 12-hour light/12-hour dark cycles for at least 2 weeks. The numbers of wheel revolution were collected every minute into a computer system. All the behavioral data were analyzed by using ClockLab software (Actimetrics). For measurement of the internal body temperature, the activity- and temperature-measuring device, nano tag (KISSEI COMTEC Co. Ltd.), was implanted into the peritoneal cavity or subcutaneous site in mice (C57BL/6 background, male, 8 weeks old). For measurement of the surface body temperature, an infrared camera (FLIR, E6) was used, and the image data were analyzed by FLIR Tools software (FLIR).

Locomotor activity rhythms of *D. melanogaster* were monitored as described previously (*35*). Male flies (2 to 5 days old) were individually housed in glass tubes (length, 65 mm; inside diameter, 3 mm) containing sucrose-agar (1% agar supplemented with 5% sucrose) food at one end and a cotton plug on the other end. The glass tubes were placed in the *Drosophila* activity monitor system (TriKinetics), and the locomotor activity of each fly was recorded as the numbers of infrared beam crossing in 1-min bin. Free-running rhythms were recorded under constant dark condition after housing under 12-hour light/12-hour dark cycles for at least 3 days. *calx^A^* or *calx^B^* mutant flies were obtained from the Bloomington *Drosophila* Stock Center.

### Mathematical analysis

By using a previously published mathematical model (*23*), we investigated an effect of CaMKII activation on the TTFL of the mammalian circadian clock. Because CaMKII phosphorylates CLOCK to activate transcriptional activity of the CLOCK-BMAL1 complex (*14*–*17*), we varied the corresponding parameter, which was represented as “phos” in the original model (*23*). Ordinary differential equations were solved numerically by using the Euler method with delta *t* = 0.001.

## SUPPLEMENTARY MATERIALS

Supplementary material for this article is available at http://advances.sciencemag.org/cgi/content/full/7/18/eabe8132/DC1

## Appendix

View/request a protocol for this paper from *Bio-protocol*.
